# A Unified and Comprehensible View of Parametric and Kernel Methods for Genomic Prediction with Application to Rice

**DOI:** 10.3389/fgene.2016.00145

**Published:** 2016-08-09

**Authors:** Laval Jacquin, Tuong-Vi Cao, Nourollah Ahmadi

**Affiliations:** Centre de Coopération Internationale en Recherche Agronomique pour le Développement, BIOS, UMR AGAPMontpellier, France

**Keywords:** genomic prediction, parametric, semi-parametric, non-parametric, kernel “trick”, epistasis

## Abstract

One objective of this study was to provide readers with a clear and unified understanding of parametric statistical and kernel methods, used for genomic prediction, and to compare some of these in the context of rice breeding for quantitative traits. Furthermore, another objective was to provide a simple and user-friendly R package, named KRMM, which allows users to perform RKHS regression with several kernels. After introducing the concept of regularized empirical risk minimization, the connections between well-known parametric and kernel methods such as Ridge regression [i.e., genomic best linear unbiased predictor (GBLUP)] and reproducing kernel Hilbert space (RKHS) regression were reviewed. Ridge regression was then reformulated so as to show and emphasize the advantage of the kernel “trick” concept, exploited by kernel methods in the context of epistatic genetic architectures, over parametric frameworks used by conventional methods. Some parametric and kernel methods; least absolute shrinkage and selection operator (LASSO), GBLUP, support vector machine regression (SVR) and RKHS regression were thereupon compared for their genomic predictive ability in the context of rice breeding using three real data sets. Among the compared methods, RKHS regression and SVR were often the most accurate methods for prediction followed by GBLUP and LASSO. An R function which allows users to perform RR-BLUP of marker effects, GBLUP and RKHS regression, with a Gaussian, Laplacian, polynomial or ANOVA kernel, in a reasonable computation time has been developed. Moreover, a modified version of this function, which allows users to tune kernels for RKHS regression, has also been developed and parallelized for HPC Linux clusters. The corresponding KRMM package and all scripts have been made publicly available.

## 1. Introduction

Since the seminal contribution of Meuwissen et al. (2001), genomic selection (GS) has become a popular strategy for genetic improvement of livestock species and plants. Moreover numerous methods from statistics and machine learning have been proposed for genomic prediction since, due to the high modeling complexity associated to the large amount of markers available. For instance, modeling the effects of thousands interacting genes (i.e., epistasis) associated to complex quantitative traits is not trivial. There is an increasing number of studies supporting that epistasis may be the most prevalent form of genetic architecture for quantitative traits (Flint and Mackay, 2009; Moore and Williams, 2009; Huang et al., 2012). Hence genomic prediction methods which can account for epistatic genetic architectures have been proposed. For example, Gianola et al. (2006) and Gianola and van Kaam (2008) first proposed reproducing kernel Hilbert space (RKHS) regression for genomic prediction when dealing with epistatic genetic architectures. Later Howard et al. (2014) showed that RKHS and support vector machine regression (SVR), when dealing with an additive genetic architecture, could be almost as competitive as parametric methods such as best linear unbiased predictor (BLUP), least absolute shrinkage and selection operator (LASSO) or Bayesian linear regressions (Bayes A, Bayes B, Bayes C, Bayes Cπ, and Bayesian LASSO). These authors also showed that RKHS regression and SVR, with some other non parametric methods, clearly outperformed parametric methods for an epistatic genetic architecture.

The SVR and kernel Ridge regression (abusively called RKHS regression in this paper with respect to previous studies (Konstantinov and Hayes, 2010; Howard et al., 2014) are popular methods known as kernel methods in the machine learning community (Cristianini and Shawe-Taylor, 2000), while they are commonly and respectively referred to as non-parametric and semi-parametric methods in statistics. Like kernel Ridge regression, SVR also performs regularization in a RKHS and this explains why kernel Ridge regression is somehow abusively called RKHS regression. Nevertheless, the term RKHS regression for kernel Ridge regression will be used in this paper so as to remain consistent with previous studies. For RKHS regression, a part of the model can be specified parametrically with fixed effects and this explains why it is also called semi-parametric regression.

There is an increasing number of studies, based on either real or simulated data, showing that kernel methods can be more appropriate than parametric methods for genomic prediction in many situations (Konstantinov and Hayes, 2010; Pérez-Rodríguez et al., 2012; Sun et al., 2012; Howard et al., 2014). In a recent review study, Morota and Gianola (2014) conjectured that RKHS regression is at least as good as linear additive models whether non-additive or additive effects are the main source of genetic variation. Their conjecture came from a series of comparison between parametric and kernel methods based on several real data sets, especially in plant breeding. The main difference between kernel and parametric methods rely in model assumptions and functional form specification. For example, SVR or RKHS regression can account for complex epistatic genetic architectures without explicitly modeling them, i.e., the model is data-driven and hence there is no pre-specified functional form relating covariates to the response (Howard et al., 2014). On the other hand classical linear regression, which is a parametric method, rely on a pre-specified functional relationship between covariates and the response.

One objective of this paper is to provide readers with a clear and unified understanding of conventional parametric and kernel methods, used for genomic prediction, and to compare some of these in the context of rice breeding for quantitative traits. Another objective is to provide an R package named KRMM which allows users to perform RKHS regression with several kernels. The first part of the paper reviews the concept of regularized empirical risk minimization as a classical formulation of learning problems for prediction. The second part reviews the equivalence between some well-known regularized linear models, such as Ridge regression and LASSO, and their Bayesian formulations. The main objective of this part is to highlight the equivalences between Ridge regression, Bayesian Ridge regression, random regression BLUP (RR-BLUP) and genomic BLUP (GBLUP), through the connections between regularized, Bayesian and mixed model regressions within the Ridge regression framework. These equivalences are important in order to understand the reformulation of Ridge regression in terms of kernel functions known as the dual formulation (Saunders et al., 1998).

In the third part we use the dual formulation of Ridge regression in order to explain and emphasize the RKHS regression methodology, in the context of epistatic genetic architectures, by the use of the so-called kernel “trick”. To our best knowledge, and according to Jiang and Reif (2015), it has not been well clarified how RKHS regression can capture multiple orders of interaction between markers and we aim at providing a simple and clear explanation to this. Jiang and Reif (2015) gave an excellent explanation on how RKHS regression, based on Gaussian kernels, can capture epistatic effects. Nevertheless, our approach is different from these authors in the sense that it is directly motivated from the kernel “trick” perspective, and hence we did not restricted ourselves to Gaussian kernels. Moreover, we used a simpler kernel function (than the Gaussian kernel) in order to give a simple and clear explanation on how RKHS regression can capture multiple orders of interaction between markers.

In the fourth part we show that solutions to many parametric and machine learning problems have similar form, due to the so-called representer theorem (Kimeldorf and Wahba, 1971), and that these solutions differ only in the choice of the loss and kernel functions used for the regularized empirical risk. We show that many parametric methods can be framed as machine learning methods with simple kernels. In the last part we compare four methods which are LASSO, GBLUP, SVR, and RKHS regression for their genomic predictive ability in the context of rice breeding using three real data sets. Finally, we provide a simple and user-friendly R function, and a tuned and parallelized version of the latter, which allow users to perform RR-BLUP of marker effects, GBLUP and RKHS regression with a Gaussian, Laplacian, polynomial or ANOVA kernel. The corresponding KRMM package and all scripts have been made publicly available at https://sourceforge.net/u/ljacquin/profile/.

## 2. Materials and methods

### 2.1. Regularized empirical risk minimization (RERM)

Here we review RERM as a classical formulation of learning problems for prediction. For simplicity reason, we consider a motivating example to RERM problems only in the linear regression framework.

#### 2.1.1. Classical formulation of RERM problems

Many statistical and machine learning problems for prediction are often formulated as follows:

(1) $$\begin{array}{c} \hat{f}(.)=\underset{f\in H}{argmin}\{\;\underset{Empirical\;risk\;term(T_{1})}{\underset{︸}{𝔼[||Y-f(X)|{|}_{2}^{2}]}}\;+\underset{\begin{array}{c} Regularization\;term(T_{2}),\\ i.e.,\;“penalty” \end{array}}{\underset{︸}{\lambda ||f|{|}_{H}}}\} \end{array}$$

where (*Y, X*) = (*Y*_*i*_, *X*_*i*_)_1≤*i*≤*n*_ are *n* independent and identically distributed (i.i.d.) data samples, according to the joint distribution of (*Y, X*), and *f* is a functional relating *Y* and *X*. H corresponds to a Hilbert space and we can take H = ℝ^*p*^ for example in the finite dimensional case, which is the Euclidean space, if *f* is a linear functional. In term *T*_2_, ||.||_H_ is a mathematical norm defined over H. For *a* ∈ H = ℝ^*p*^, we can define $||a|{|}_{H}=||a|{|}_{q}={\left(\overset{p}{\underset{i=1}{\sum }}|a_{i}{|}^{q}\right)}^{\frac{1}{q}}$ which is the *L*^*q*^ norm for example. In Expression (1) $\hat{f}\left(.\right)$ corresponds to a functional (i.e., “model”) minimizing simultaneously *T*_1_ and *T*_2_ over H. Note that the uniqueness of $\hat{f}\left(.\right)$ depends on the norm used in *T*_2_ and the sizes of *n* and *p*. Term *T*_2_ is called the regularization (or penalization) term which has a tuning parameter λ controlling the “size” of *f* (i.e., model complexity). Term *T*_1_ is called the empirical risk and corresponds, for some loss function, to the expected (i.e., 𝔼[.] ) data prediction error which can be estimated using the empirical mean by the weak law of large numbers (Cornuéjols and Miclet, 2011). A common choice for the loss function is the squared *L*^2^ norm (i.e., $||.|{|}_{q}^{2}$ with *q* = 2), even though other choices such as the *L*^1^ norm, or the ε-insensitive loss like in the case of SVR (Smola and Schlkopf, 1998), are possible. Finally, finding the solution to Expression (1) is known as a RERM problem.

#### 2.1.2. A motivating example for RERM problems

Here we review the motivation behind RERM problems within the classical linear regression framework for the sake of simplicity. Assume that we have a functional relationship *Y* = f^*^(*X*) + ε^*^, where *Y* = [*Y*_1_, .., *Y*_*i*_, .., *Y*_*n*_] is a vector of *n* measured phenotypic responses, *X* = (*X*_*i*_)_1≤*i*≤*n*_ is an *n* x *p* marker genotype matrix with $X_{i}=\left[X_{i}^{\left(1\right)},X_{i}^{\left(2\right)},..,X_{i}^{\left(j\right)},..,X_{i}^{\left(p\right)}\right]\in {\mathbb{R}}^{p}$ (i.e., genotypes at *p* markers for individual *i*) and $\varepsilon^{*}={\left[\varepsilon_{1}^{*},\varepsilon_{2}^{*},..,\varepsilon_{i}^{*},..,\varepsilon_{n}^{*}\right]}^{\prime }$ is the error vector of *n* i.i.d elements with $𝔼\left[\varepsilon_{i}^{*}\right]=0$ and $𝕍ar\left[\varepsilon_{i}^{*}\right]=\sigma_{\varepsilon^{*}}^{2}>0$, where $\sigma_{\varepsilon^{*}}^{2}$ is unknown. f^*^(.) can be interpreted as the “true” deterministic model, or the data generating process (DGP), generating the true genetic values of individuals. Note that we do not assume gaussianity for ε^*^ here. Our aim is to identify a model with linear regression that best approximates f^*^(.). Consider the following linear model with full rank *X* (⇒ *p* ≤ *n*):

(2) $$\begin{array}{l} Y_{i}=\beta_{1}X_{i}^{\left(1\right)}+\beta_{2}X_{i}^{\left(2\right)}+\beta_{3}X_{i}^{\left(3\right)}+\ldots +\beta_{j}X_{i}^{\left(j\right)}+\ldots +\beta_{p}X_{i}^{\left(p\right)}\\ \;+\;\varepsilon_{i}=f_{p}\left(X_{i}\right)+\varepsilon_{i}\text{ where }f_{p}\left(X_{i}\right)=\overset{p}{\underset{j=1}{\sum }}\beta_{j}X_{i}^{\left(j\right)} \end{array}$$

In matrix notation we can write the model defined by Equation (2) as *Y* = *f*_*p*_(*X*) + ε = *Xβ* + ε where $\beta ={\left[\beta_{1},\beta_{2},..,\beta_{j},..,\beta_{p}\right]}^{\prime }$. By ordinary least squares (OLS), the estimated model for Equation (2) is given by ${\hat{f}}_{p}\left(X\right)=X{\hat{\beta }}_{OLS}$, where ${\hat{\beta }}_{OLS}$ is the unique minimizer of $||Y-X\beta |{|}_{2}^{2}=||\varepsilon |{|}_{2}^{2}$ (which is strictly convex and quadratic) and is given by ${\hat{\beta }}_{OLS}={\left(X^{\prime }X\right)}^{-1}X^{\prime }Y$. For this estimated model we have the following property which holds (see Supplementary Material, lemma 4):

(3) $$\begin{array}{l} \underset{\begin{array}{c} \text{Risk of the model},\text{ i}.\text{e}.,\text{ distance}\\ \text{between estimated model}\\ \text{and true model }\left(R_{1}\right) \end{array}}{\underset{︸}{𝔼\left[\;||{\hat{f}}_{p}\left(X\right)-f^{*}\left(X\right)|{|}_{2}^{2}\;\right]}}=\underset{\begin{array}{c} \text{Empirical risk term }\left(R_{2}\right) \end{array}}{\underset{︸}{𝔼\left[\;||Y-{\hat{f}}_{p}\left(X\right)|{|}_{2}^{2}\;\right]}}\\ \;+\underset{\begin{array}{c} \text{Term with dependence}\\ \text{on number of}\\ \text{parameters }\left(R_{3}\right) \end{array}}{\underset{︸}{2\sigma_{\varepsilon^{*}}^{2}p}}\underset{\left(R_{4}\right)}{\underset{︸}{-\sigma_{\varepsilon^{*}}^{2}n}}\; \end{array}$$

For a fixed sample size *n*, from Equation (3) we clearly see that *R*_2_ → 0 and *R*_3_ → +∞ when *p* → +∞. This situation is common in genomic prediction where *p* is often much bigger than *n*, i.e., *p* >> *n*. Precisely, we have *R*_2_ = 0 (i.e., $Y={\hat{f}}_{p}\left(X\right)$) when *p* ≥ *n*. This is due to the fact that ${\hat{f}}_{p}\left(X\right)$ is the orthogonal projection of *Y* on the subspace of ℝ^*n*^, generated by columns of *X*, which becomes ℝ^*n*^ when *p* ≥ *n* (see Supplementary Material, lemma 5). This phenomena is a case of what is known as overfitting since the estimated model reproduces the data, which contains the error term, and is not describing the underlying relation defined by f^*^(.). Note that *R*_4_ is unaffected by *p* for a fixed *n*. Hence, if we want to decrease the distance between the estimated model and the true model (i.e., *R*_1_), we need to minimize simultaneously *R*_2_ and *R*_3_ and this motivates the RERM formulation seen in Equation (1). Note that minimizing *R*_3_ (i.e., decreasing *p*), with *R*_2_ simultaneously, will penalize model complexity, and size, and this explains why a regularization term is also called a penalization term.

### 2.2. Equivalence of regularized and Bayesian formulations of Ridge and LASSO regressions

In what follows, we assume that matrix *X* and vector *Y* are centered. Two popular examples of regularized linear regressions are Ridge regression (Hoerl and Kennard, 1970) and LASSO (Tibshirani, 1996). These (estimated) models and their regularized estimates are given by:

(4) $$\begin{array}{l} \hat{f}{\left(X\right)}_{Ridge}=X{\hat{\beta }}_{Ridge}\\ \text{where }{\hat{\beta }}_{Ridge}=\underset{\beta \in {\mathbb{R}}^{p}}{argmin}\left\{\;||Y-X\beta |{|}_{2,{\mathbb{R}}^{n}}^{2}+\lambda ||\beta |{|}_{2,{\mathbb{R}}^{p}}^{2}\;\right\} \end{array}$$

(5) $$\begin{array}{l} ={\left(X^{\prime }X+\lambda I_{p}\right)}^{-1}X^{\prime }Y \end{array}$$

(6) $$\begin{array}{l} \hat{f}{\left(X\right)}_{LASSO}=X{\hat{\beta }}_{LASSO}\\ \text{where }{\hat{\beta }}_{LASSO}=\underset{\beta \in {\mathbb{R}}^{p}}{argmin}\left\{\;||Y-X\beta |{|}_{2,{\mathbb{R}}^{n}}^{2}+\lambda ||\beta |{|}_{1,{\mathbb{R}}^{p}}\;\right\} \end{array}$$

Note that ${\hat{\beta }}_{LASSO}$ does not admit a closed form like ${\hat{\beta }}_{Ridge}$ in the general case. Indeed the *L*^1^ norm in the LASSO penalty makes the objective function non-differentiable when β_*j*_ = 0 for any β_*j*_. However, a closed form for ${\hat{\beta }}_{LASSO}$ is available via the *soft-thresholding operator* and the OLS estimate when *X* is orthonormal (i.e., *X*′*X* = *XX*′ = *I*), which is however rarely the case with SNP markers. Nevertheless, there are many possible algorithms to compute LASSO solutions such as the least angle regression selection (LARS) (Efron et al., 2004), proximal gradient descent based iterative soft-thresholding (ISTA) (Gordon and Tibshirani, 2012), cyclic coordinate descent (Friedman et al., 2010) and etc. However, these algorithms are beyond the scope of this article and are not the focus here. The objective functions in Problem (4) and (6) are also particular type of functions called relaxed Lagrangians, which are unconstrained formulations of constrained optimization problems. Searching for the saddle points of these Lagrangians is equivalent to searching for the solutions to the constrained formulations of Problem (4) and (6). Specifically, the solutions to Problem (4) and (6) are obtained when the ellipses, defined by the contour lines of the empirical risk term, touch the different constrained regions for β imposed by the *L*^2^ and *L*^1^ norms respectively. Hence, the *L*^1^ norm generally induces sparsity in the LASSO solution, compared to the *L*^2^ norm which induces a shrinkage of the β_*j*_ in the Ridge solution, when λ increases (Friedman et al., 2001).

Another way to tackle Problem (4) and (6) is in a probabilistic manner via a Bayesian treatment. Moreover the Bayesian treatment allows one to see the direct equivalence between Ridge regression, Bayesian Ridge regression, RR-BLUP and GBLUP. The equivalence between Ridge regression, RR-BLUP and GBLUP is a direct consequence of the proof of equivalence between Ridge regression and Bayesian Ridge regression (Lindley and Smith, 1972; Bishop and Tipping, 2003; De los Campos et al., 2013a). The proof found in De los Campos et al. (2013a) is reported below.

*Proof of equivalence between Ridge regression and Bayesian Ridge regression:*

(7) $$\begin{array}{l} {\hat{\beta }}_{Ridge}=\underset{\beta \in {\mathbb{R}}^{p}}{argmin}\left\{{\sum_{i\;=\;1}^{n}\left[Y_{i}-\sum_{j\;=\;1}^{p}X_{i}^{\left(j\right)}\beta_{j}\right]}^{2}+\lambda \sum_{j\;=\;1}^{p}\beta_{j}^{2}\right\}\\ \;(\text{take }\lambda \text{=}\frac{\sigma_{\varepsilon }^{2}}{\sigma_{\beta }^{2}}\text{ and }\times -\frac{1}{2}) \end{array}$$

(8) $$=\underset{\beta \in {\mathbb{R}}^{p}}{argmax}\left\{-\frac{1}{2}{\sum_{i\;=\;1}^{n}\left[Y_{i}-\sum_{j\;=\;1}^{p}X_{i}^{\left(j\right)}\beta_{j}\right]}^{2}-\frac{1}{2}\frac{\sigma_{\varepsilon }^{2}}{\sigma_{\beta }^{2}}\sum_{j=1}^{p}\beta_{j}^{2}\right\}$$

(9) $$\begin{array}{l} (\text{divide by }\sigma_{\varepsilon }^{\text{2}}\text{ and apply monotonic transformation }e^{x})\text{ ​}\\ =\text{​}\underset{\beta \in {\mathbb{R}}^{p}}{\;argmax}=\underset{i.e.,\;Y|\beta ,\sigma_{\varepsilon }^{2}\sim N_{n}(X\beta ,I_{n}\sigma_{\varepsilon }^{2})}{\underset{︸}{\{\prod_{i\;=\;1}^{n}N(Y_{i}|\sum_{j\;=\;1}^{p}X_{i}^{\left(j\right)}\beta_{j},\sigma_{\varepsilon }^{2})}}\text{​ }\times \text{ ​}\underset{i.e.,\;\beta |\sigma_{\beta }^{2}\sim N_{p}(0,I_{p}\sigma_{\beta }^{2})}{\underset{︸}{\overset{p}{\underset{j\;=\;1}{\prod }}N(\beta_{j}|0,\sigma_{\beta }^{2})\}}} \end{array}$$

(10) $$\begin{array}{l} =\underset{\beta \in {\mathbb{R}}^{p}}{argmax}\left\{f(\beta |Y,\sigma_{\varepsilon }^{2},\sigma_{\beta }^{2})\right\}=\text{mode }\left\{f(\beta |Y,\sigma_{\varepsilon }^{2},\sigma_{\beta }^{2})\right\}\\ ={\hat{\beta }}_{Bayesian\;Ridge} \end{array}$$

where $f\left(\beta |Y,\sigma_{\varepsilon }^{2},\sigma_{\beta }^{2}\right)$ is the density of the posterior distribution for β (i.e., marker effects) in (10). Due to the proportionality between the posterior density, and the product of gaussian densities for the likelihood and the prior distribution for β, $f\left(\beta |Y,\sigma_{\varepsilon }^{2},\sigma_{\beta }^{2}\right)$ is also the density of a gaussian distribution by conjugacy. Thus, by symmetry of the gaussian distribution for $\beta |Y,\sigma_{\varepsilon }^{2},\sigma_{\beta }^{2}$, we have $\text{mode}\{f\left(\beta |Y,\sigma_{\varepsilon }^{2},\sigma_{\beta }^{2})\right\}$ $=𝔼\left(\beta |Y,\sigma_{\varepsilon }^{2},\sigma_{\beta }^{2}\right)=\mathbb{C}ov\left(\beta ,Y\right)𝕍ar{\left(Y\right)}^{-1}Y$ $=I_{p}\sigma_{\beta }^{2}X^{\prime }{\left[XX^{\prime }\sigma_{\beta }^{2}+\sigma_{\varepsilon }^{2}I_{n}\right]}^{-1}Y$ = $X^{\prime }{\left[XX^{\prime }+\lambda I_{n}\right]}^{-1}Y$ under the assumption that ℂ*ov*(β, ε) = 0, where $𝔼\left(\beta |Y,\sigma_{\varepsilon }^{2},\sigma_{\beta }^{2}\right)$ can be identified to be the BLUP (Robinson, 1991; Schaeffer, 2010) of β and corresponds to the solution of the RR-BLUP model: ${\hat{\beta }}_{RR-BLUP}=X^{\prime }{\left[XX^{\prime }+\lambda I_{n}\right]}^{-1}Y$. Hence we have ${\hat{\beta }}_{Ridge}={\hat{\beta }}_{Bayesian\;Ridge}={\hat{\beta }}_{RR-BLUP}$.

We recall that the RR-BLUP model (Ruppert et al., 2003) corresponds to the following mixed model *Y* = *Xβ* + ε, where $\beta \sim N_{p}\left(0,I_{p}\sigma_{\beta }^{2}\right)$, $\varepsilon \sim N_{n}\left(0,I_{n}\sigma_{\varepsilon }^{2}\right)$ and ℂ*ov*(β, ε) = 0. If *U* = *Xβ*, this model can be rewritten as *Y* = *U* + ε, where $U\sim N_{n}\left(0,\sigma_{U}^{2}𝕌\right)$ with genomic covariance matrix 𝕌 = *XX*′ and $\sigma_{U}^{2}=\sigma_{\beta }^{2}$. The GBLUP of *U* for this model is given by Û = ℂ*ov*(*U, Y*)𝕍*ar*(*Y*)^−1^*Y* = $XX^{\prime }{\left[XX^{\prime }+\lambda I_{n}\right]}^{-1}Y$. So it is clear that predictions obtained with RR-BLUP and GBLUP are mathematically equivalent.

The equivalence between LASSO and Bayesian LASSO, i.e., ${\hat{\beta }}_{LASSO}={\hat{\beta }}_{Bayesian\;LASSO}$, can be shown using the same type of arguments as for the proof of equivalence between Ridge regression and Bayesian Ridge regression. For example, the proof of equivalence between LASSO and Bayesian LASSO can also be found in De los Campos et al. (2013a). In the case of Bayesian LASSO the prior density for β corresponds to the product of *p* i.i.d Laplace densities for the marker effects (Tibshirani, 1996; De los Campos et al., 2013a). Thus, the prior distributions for β, in Bayesian Ridge regression and Bayesian LASSO, give another insight on the shrinked and sparse solutions for Ridge and LASSO respectively.

### 2.3. Dual formulation of Ridge regression in terms of kernel functions

We recall that the classical formulation of Ridge regression is given by $\hat{f}{\left(X\right)}_{Ridge}=X{\hat{\beta }}_{Ridge}$ where ${\hat{\beta }}_{Ridge}={\left(X^{\prime }X+\lambda I_{p}\right)}^{-1}X^{\prime }Y$. This formulation is also known as the primal formulation of Ridge regression. However, one can notice that the Ridge solution can be written as ${\hat{\beta }}_{Ridge}=X^{\prime }{\hat{\alpha }}^{Ridge}$ where ${\hat{\alpha }}^{Ridge}=\frac{1}{\lambda }\left[Y-X{\hat{\beta }}_{Ridge}\right]$. Therefore, by substituting $X^{\prime }{\hat{\alpha }}^{Ridge}$ for ${\hat{\beta }}_{Ridge}$ in the expression for ${\hat{\alpha }}^{Ridge}$, we also have ${\hat{\alpha }}^{Ridge}={\left(XX^{\prime }+\lambda I_{n}\right)}^{-1}Y$. Hence Ridge regression can be reformulated as follows:

(11) $$\hat{f}{\left(X\right)}_{Ridge}=XX\prime {\hat{\alpha }}^{Ridge}\text{ where }{\hat{\alpha }}^{Ridge}={\left(XX\prime +\lambda I_{n}\right)}^{-1}Y\in {\mathbb{R}}^{\text{n}}$$

Expression (11) is called the dual formulation of Ridge regression (Saunders et al., 1998), where the components of the vector ${\hat{\alpha }}^{Ridge}=\left({\hat{\alpha }}_{1}^{Ridge},{\hat{\alpha }}_{2}^{Ridge},\ldots .,{\hat{\alpha }}_{n}^{Ridge}\right)$ are called dual variables. It is clear that Expression (11) is identical to the GBLUP expression seen in the previous section. Hence, the classical formulation of Ridge regression and GBLUP are primal and dual formulations, respectively, of the same solution to a RERM problem. Note that Expression (11) requires the inversion of an *n* × *n* matrix compared to Expression (5) where a *p* × *p* matrix needs to be inverted. This is particularly convenient in the context of SNP markers where *p* >> *n*. If we let 𝕂 = *XX*′, Expression (11) can be written more conveniently as:

(12) $$\hat{f}{\left(X\right)}_{Ridge}=𝕂{\hat{\alpha }}^{Ridge}\text{ where }{\hat{\alpha }}^{Ridge}={\left(𝕂+\lambda I_{n}\right)}^{-1}Y$$

For each genotype vector $X_{i}=\left[X_{i}^{\left(1\right)},X_{i}^{\left(2\right)},..,X_{i}^{\left(j\right)},..,X_{i}^{\left(p\right)}\right]\in {\mathbb{R}}^{p}$, Expression (12) can be written as:

(13) $$\hat{f}{\left(X_{i}\right)}_{Ridge}=\sum_{j\;=\;1}^{n}{\hat{\alpha }}_{j}^{Ridge}{𝕂}_{ij}=\sum_{j\;=\;1}^{n}{\hat{\alpha }}_{j}^{Ridge}<X_{i},X_{j}{>}_{{\mathbb{R}}^{p}}$$

where ${𝕂}_{ij}=\;<X_{i},X_{j}{>}_{{\mathbb{R}}^{p}}$ are elements of 𝕂, i.e., 𝕂 = (𝕂_*ij*_)_1≤*i,j*≤*n*_, and $<.,.{>}_{{\mathbb{R}}^{p}}$ denotes the inner product between two vectors in ℝ^*p*^. Expression (13) is particularly helpful as it can allow one to understand the kernel “trick” exploited by kernel methods, in the context of epistatic genetic architectures, as shown by the following example.

Consider the school case where we have *p* = 2 markers, i.e., $X_{i}=\left[\;X_{i}^{\left(1\right)},\;X_{i}^{\left(2\right)}\;\right]$, and *n* measured phenotypic responses. Moreover, consider the following transformation ϕ applied to *X*_*i*_: $\phi \left(X_{i}\right)=\left[\;{\left(X_{i}^{\left(1\right)}\right)}^{2},\underset{Interaction\;term}{\underset{︸}{\sqrt{2}X_{i}^{\left(1\right)}X_{i}^{\left(2\right)}}},{\left(X_{i}^{\left(2\right)}\right)}^{2}\;\right]=\left[\;\phi^{\left(1\right)}\left(X_{i}\right),\;\phi^{\left(2\right)}\left(X_{i}\right),\;\phi^{\left(3\right)}\left(X_{i}\right)\;\right]\in {\mathbb{R}}^{3}$ where $\phi^{\left(2\right)}\left(X_{i}\right)$ corresponds to the interaction term between $X_{i}^{\left(1\right)}$ and $X_{i}^{\left(2\right)}$. In what follows we define ϕ(*X*) to be the *n* × 3 transformed marker genotype matrix. Hence, for our school case, two possible models for example are given by:

(14) $$\begin{array}{l} Model\;1\;(M_{1})\text{​}:\hat{f}(X_{i})={\hat{\beta }}_{1}X_{i}^{\left(1\right)}+{\hat{\beta }}_{2}X_{i}^{\left(2\right)}\\ \text{ where }\left(\begin{array}{c} {\hat{\beta }}_{\text{1}}\\ {\hat{\beta }}_{\text{2}} \end{array}\right)=\underset{\beta \in {\mathbb{R}}^{2}}{argmin}\{\;||Y-X\beta |{|}_{2,{\mathbb{R}}^{n}}^{2}+\lambda ||\beta |{|}_{2,{\mathbb{R}}^{2}}^{2}\;\}\\ \;\Leftrightarrow \hat{f}(X_{i})=\sum_{j=1}^{n}{\hat{\alpha }}_{j}^{M_{1}}{𝕂}_{ij}=\sum_{j=1}^{n}{\hat{\alpha }}_{j}^{M_{1}}<X_{i},X_{j}{>}_{{\mathbb{R}}^{2}} \end{array}$$

(15) $$\begin{array}{l} Model\;2\;(M_{2})\text{​}:\hat{f}(X_{i})={\hat{\theta }}_{1}\phi^{\left(1\right)}(X_{i})+{\hat{\theta }}_{2}\phi^{\left(2\right)}(X_{i})+{\hat{\theta }}_{3}\phi^{\left(3\right)}(X_{i})\\ \text{ where }\left(\begin{array}{c} {\hat{\theta }}_{\text{1}}\\ {\hat{\theta }}_{\text{2}}\\ {\hat{\theta }}_{\text{3}} \end{array}\right)=\underset{\theta \in {\mathbb{R}}^{3}}{argmin}\{\;||Y-\phi (X)\theta |{|}_{2,{\mathbb{R}}^{n}}^{2}+\lambda ||\theta |{|}_{2,{\mathbb{R}}^{3}}^{2}\;\}\\ \;\Leftrightarrow \hat{f}(X_{i})=\sum_{j\;=\;1}^{n}{\hat{\alpha }}_{j}^{M_{2}}{𝕂}_{ij}^{\phi }=\sum_{j\;=\;1}^{n}{\hat{\alpha }}_{j}^{M_{2}}<\phi (X_{i}),\phi (X_{j}){>}_{{\mathbb{R}}^{3}}\\ \text{ where }{𝕂}^{\phi }=\;\phi (X)\phi {\left(X\right)}^{\prime }\text{ and }{\hat{\alpha }}^{{\text{M}}_{2}}={\left({𝕂}^{\phi }+\lambda I_{n}\right)}^{-1}Y \end{array}$$

However one can notice that ${𝕂}_{ij}^{\phi }=\;<\phi \left(X_{i}\right),\phi \left(X_{j}\right){>}_{{\mathbb{R}}^{3}}\;={\left(<X_{i},X_{j}{>}_{{\mathbb{R}}^{2}}\right)}^{2}={\left({𝕂}_{ij}\right)}^{2}$. Indeed we have:

$$\begin{array}{l} <\phi (X_{i}),\phi (X_{j}){>}_{{\mathbb{R}}^{3}}=[{\left(X_{i}^{\left(1\right)}X_{j}^{\left(1\right)}\right)}^{2}+2(X_{i}^{\left(1\right)}X_{j}^{\left(1\right)})(X_{i}^{\left(2\right)}X_{j}^{\left(2\right)})\\ \;+{\left(X_{i}^{\left(2\right)}X_{j}^{\left(2\right)}\right)}^{2}]\\ \;={\left[X_{i}^{\left(1\right)}X_{j}^{\left(1\right)}+X_{i}^{\left(2\right)}X_{j}^{\left(2\right)}\right]}^{2}={\left(<X_{i},X_{j}{>}_{{\mathbb{R}}^{2}}\right)}^{2} \end{array}$$

This means that we only need to square the elements of matrix 𝕂 for *Model* 1 to obtain *Model* 2 (i.e., to perform a Ridge regression in ℝ^3^ modeling an interaction term). Indeed, in matrix form *Model* 2 can be written as:

(16) $$\hat{f}{\left(X\right)}_{Ridge}={𝕂}^{\phi }{\left({𝕂}^{\phi }+\lambda I_{n}\right)}^{-1}Y\text{ where }{𝕂}_{ij}^{\phi }={\left({𝕂}_{ij}\right)}^{2}$$

Similarly, for the case of *p* = 3 markers we can perform a Ridge regression in ℝ^6^, which models three interaction terms, by just squaring the inner product between genotype vectors in ℝ^3^, i.e., ${𝕂}_{ij}^{\phi }={\left(<X_{i},X_{j}{>}_{{\mathbb{R}}^{3}}\right)}^{2}$. This process of implicitly computing inner products, in the space of transformed genotype vectors, by performing computations only in the original space of genotype vectors is known as the kernel “trick.” The space of transformed covariates (i.e., space of transformed genotype vectors here), associated to a map ϕ, is commonly known as a feature space in machine learning. A kernel function associated to a feature map ϕ is defined as follows.

#### Definition of a kernel k:

For *X*_*i*_, *X*_*j*_ ∈ *E*, a kernel *k* is a function which satisfies *k*(*X*_*i*_, *X*_*j*_) = < ϕ(*X*_*i*_), ϕ(*X*_*j*_) >_*F*_, where *E* and *F* are the space of covariates and feature space respectively.

For example, in our school case we used the quadratic kernel defined by $k\left(X_{i},X_{j}\right)={\left(<X_{i},X_{j}{>}_{E}\right)}^{2}=\;<\phi \left(X_{i}\right),\phi \left(X_{j}\right){>}_{F}$ where *F* = ℝ^3^ when *E* = ℝ^2^ (i.e., *p* = 2 markers). Note that there is no one-to-one correspondence between a feature map ϕ and a kernel *k*. Indeed, more that one feature map can be associated to a unique kernel (see Supplementary Material, lemma 6). In classical Ridge regression we do not have any interaction term and the feature map is the identity (i.e., ϕ = *id*) since 𝕂_*ij*_ = *k*(*X*_*i*_, *X*_*j*_) = < *X*_*i*_, *X*_*j*_ > in this situation. A necessary and sufficient condition for a function *k* to be a kernel is that matrix 𝕂 = *k*(*X*_*i*_, *X*_*j*_)_1≤*i,j*≤*n*_ (known as the Gram matrix) is positive semi-definite. This condition comes from Mercer's theorem (Gretton, 2013) and it gives a practical way to check if a function *k* defines a kernel.

Some kernels are called universal kernels in the sense that they can approximate any arbitrary function f^*^(.), with a finite number of training samples, if regularized properly (Micchelli et al., 2006). One such example is the Gaussian kernel given by $k\left(X_{i},X_{j}\right)=e^{-h||X_{i}-X_{j}|{|}_{2}^{2}}$, where *h* > 0 is a rate of decay parameter for *k*. This kernel is associated to an infinite-dimensional feature map which allows an implicit modeling of all possible orders of interaction between markers (see Supplementary Material, lemma 7). Hence, the Gaussian kernel is useful for genomic prediction when dealing with complex epistatic genetic architectures.

### 2.4. RKHS and the representer theorem

The concept of RKHS (Smola and Schlkopf, 1998; Cornuéjols and Miclet, 2011; Gretton, 2013) with its implications in statistics and machine learning are well beyond the scope of this article. Here we review the basic definition of a RKHS so as to introduce the representer theorem which exploits the definition of RKHS. The representer theorem has important applications in practice. Indeed, it can allow one to find optimal solution to RERM problems and it shows that solutions to many parametric and machine learning problems have similar form.

#### Definition of a RKHS:

Let ϕ(*X*_*i*_) = *k*(., *X*_*i*_), a RKHS *H*_*k*_ associated to a kernel *k* can be defined as a space of functions generated by linear combinations of *k*(., *X*_*i*_);

$$H_{k}=\left\{\sum_{i\;=\;1}^{n}\alpha_{i}k(.,X_{i});\;X_{i}\in E,\;\alpha_{i}\in \mathbb{R},\;n\in \mathbb{N}\right\}$$

such that (i) for all *X*_*i*_ ∈ *E, k*(., *X*_*i*_) ∈ *H*_*k*_ and (ii) for all *X*_*i*_ ∈ *E* and every *f*(.) ∈ *H*_*k*_, < *f*(.), *k*(., *X*_*i*_)>_*H*_*k*__ = *f*(*X*_*i*_) (Cornuéjols and Miclet, 2011). The condition (ii) is called the reproducing property of *k* as it reproduces *f* in some sense. Hence, from the reproducing property we have < ϕ(*X*_*i*_), ϕ(*X*_*j*_) >=< *k*(., *X*_*i*_), *k*(., *X*_*j*_) >= *k*(*X*_*i*_, *X*_*j*_). According to Moore-Aronszajn theorem, every RKHS has a unique positive semi-definite kernel (i.e., a reproducing kernel) and vice-versa. In other words, there is one-to-one correspondence between RKHS and kernels. A simplified version of the representer theorem is given as follows.

#### The representer theorem (Kimeldorf and Wahba, 1971):

Fix a set *E* and a kernel *k*, and let *H*_*k*_ be the corresponding RKHS. For any loss function *L*:ℝ^2^ → ℝ, the solution $\hat{f}$ of the optimization problem;

(17) $$\hat{f}(.)=\underset{f\in H_{k}}{argmin}\{\;\sum_{i\;=\;1}^{n}L(Y_{i},f(X_{i}))\;+\lambda ||f|{|}_{H_{k}}^{2}\}$$

has the following form:

(18) $$\hat{f}(.)=\sum_{i\;=\;1}^{n}\alpha_{i}k(.,X_{i})$$

This result is of great practical importance. For example, if we substitute the representation Equation (18) into Equation (17) when $L\left(Y_{i},f\left(X_{i}\right)\right)={\left(\;Y_{i}-f\left(X_{i}\right)\;\right)}^{2}$ (aka kernel Ridge regression) then we obtain the following equivalent problem;

(19) $${\hat{\alpha }}_{Kernel\;Ridge}=\underset{\alpha \in {\mathbb{R}}^{n}}{argmin}\{\;\frac{1}{2}||Y-𝕂\alpha |{|}_{2}^{2}+\frac{\lambda }{2}\alpha \prime 𝕂\alpha \;\}$$

where ${\hat{\alpha }}_{Kernel\;Ridge}$ can be shown to be given by ${\hat{\alpha }}_{Kernel\;Ridge}={\left[𝕂+\lambda I_{n}\right]}^{-1}Y$. Moreover, if we follow the same reasoning as for Equation (7) to Equation (10), one can easily show from Equation (19) that ${\hat{\alpha }}_{Kernel\;Ridge}={\hat{\alpha }}_{Bayesian\;Kernel\;Ridge}={\hat{\alpha }}_{RR-BLUP}$, where ${\hat{\alpha }}_{RR-BLUP}$ is the BLUP of α for the following mixed model;

$$\begin{array}{l} Y=𝕂\alpha +\varepsilon \text{ where }\alpha \sim N_{n}(0,\sigma_{\alpha }^{2}{𝕂}^{-1})\text{ and }\varepsilon \sim N_{n}(0,\sigma_{\varepsilon }^{2})\\ \Leftrightarrow Y=g+\varepsilon \text{ where }g=𝕂\alpha \sim N_{n}(0,\sigma_{g}^{2}𝕂)\text{ , with }\sigma_{g}^{2}=\sigma_{\alpha }^{2}\text{ ,}\\ \text{ and }\varepsilon \sim N_{n}(0,\sigma_{\varepsilon }^{2}) \end{array}$$

Hence, the mixed model methodology can be used to solve kernel Ridge regression (i.e., RKHS regression) for which classical Ridge regression (i.e., GBLUP) is a particular case.

In Expression (17) we have *L*(*Y*_*i*_, *f*(*X*_*i*_)) = |*Y*_*i*_ − *f*(*X*_*i*_)|_ε_ for SVR (i.e., ε-insensitive loss), proposed by Vapnik (1998), which is given by:

$$|Y_{i}-f(X_{i}){|}_{\varepsilon }=\{\begin{array}{ll} 0 & \text{ if }|Y_{i}-f(X_{i})|\;\leq \varepsilon \\ |Y_{i}-f(X_{i})|-\varepsilon & \text{ otherwise } \end{array}$$

Note that SVR also performs regularization in a RKHS. The parameter λ in Equation (17) correspond to $\frac{1}{2C}$ (where *C* > 0) in the original formulation of SVR (Basak et al., 2007). Moreover, slack variables are also found in the original formulation in order to cope with infeasible constraints. SVR has several interesting properties. For example, the dual optimization problem of finding the Lagrange multipliers (i.e., dual variables: $\alpha_{j},\alpha_{j}^{*}$) in SVR, and in support vector machine for classification, is generally a constrained Quadratic Programming (QP) problem which assures a global minimum. Furthermore, only a fraction of the Lagrange multipliers are non-zero, due to the so-called Karush-Kuhn-Tucker (KKT) conditions which state that the product between dual variables and constraints vanishes at the optimal solution. The genotype vectors (i.e., *X*_*j*_) corresponding to non-zero Lagrange multipliers are called support vectors as they are the only ones which contribute to prediction. This is particularly convenient for data sets with a large number of accessions where we need only the support vectors for prediction. Indeed, the estimated prediction function in SVR can we written as;

$$\hat{f}{\left(X_{i}\right)}_{SVR}=\sum_{j\;=\;1}^{n}{\bar{\alpha }}_{j}k(X_{i},X_{j})+b\text{ with }{\bar{\alpha }}_{j}=({\hat{\alpha }}_{j}-{\hat{\alpha }}_{j}^{*})\text{ and }b\in \mathbb{R}$$

where only a restricted number of ${\bar{\alpha }}_{j}$ are non-zero and have corresponding support vectors *X*_*j*_ (note that both ${\hat{\alpha }}_{j}$ and ${\hat{\alpha }}_{j}^{*}$ cannot be non-zero simultaneously Basak et al., 2007; Al-Anazi and Gates, 2012). These vectors are associated to approximation errors which are greater than ε. Thus, the number of support vectors is inversely proportional to the ε parameter. Further details on SVR, and on support vector machine in the general case, can be found in Vapnik (1998), Smola and Schlkopf (1998), Basak et al. (2007), and Al-Anazi and Gates (2012). Finally, note that we do not have the representation Equation (18) for LASSO since the *L*^1^ norm, for this particular case, violates the representer theorem assumptions.

### 2.5. Analyzed data sets and prediction methods compared

Three real data sets were analyzed. The first data set was composed of 230 temperate japonica accessions with 22,691 SNP. For the second data set, 167 tropical japonica accessions with 16,444 SNP were available. The third data set was composed of 188 tropical japonica accessions with 38,390 SNP. A total of 15 traits were analyzed for the three data sets. Plant height (PH), flowering time (FL), leaf arsenic content (AR), number of tillers (NT), shoot biomass (SB), maximum root length (RL), number of roots below 30 centimeters (NR), deep root biomass (DR) and root over shoot biomass ratio (RS) were analyzed for the first and second data sets. For the third data set, PH, cycle duration (CD), fertility rate (FE), number of seeds per panicle (NS), straw yield in kilograms per hectare (SY) and number of panicles per square metre (NP) were analyzed. All SNP marker data sets had a minor allele frequency strictly superior to 1%. The three data sets are officially available at http://tropgenedb.cirad.fr/tropgene/JSP/interface.jsp?module=RICE as the “GS-RUSE.zip" folder, or can be downloaded directly at http://tropgenedb.cirad.fr/tropgene/downloads/studies/GS-RUSE.zip.

Four methods; LASSO, GBLUP, RKHS regression and SVR were applied to these data sets and traits, and hence a total of 60 situations were examined. R scripts were written to perform analyses with the four methods and are available on request. The glmnet (Friedman et al., 2010) and kernlab (Karatzoglou et al., 2004) packages were used for LASSO and SVR respectively. R scripts were written to solve GBLUP and RKHS regression. The expectation-maximization (EM) algorithm (Dempster et al., 1977; Foulley, 2002; Jacquin et al., 2014) was used to maximize the restricted likelihoods (REML) of the mixed models, associated to GBLUP and RKHS regression respectively, in order to estimate the associated variance parameters. The Gaussian kernel was used for RKHS regression and SVR. For RKHS regression, values over several grids were tested using cross-validation to tune the rate of decay parameter for each data set. For SVR, the rate of decay was estimated using the heuristic defined in the sigest function (Karatzoglou et al., 2004), which is already implemented in the ksvm function (Karatzoglou et al., 2004), that allows an automatic selection of this parameter. The regularization parameter *C* for SVR was estimated as *C* = *max*(|*Y* + 3σ_*Y*_|, |*Y* − 3σ_*Y*_|), where *Y* is the phenotypic mean, as recommended by Cherkassky and Ma (2004). Values higher than 0.5 for the ε parameter in SVR produced no support vectors. Hence lower values were tested for this parameter using cross validation. Values ranging between 0.01 and 0.1 were found to give similar and the best predictive performance for each data set, hence ε was fixed to 0.01. For LASSO, the cv.glmnet function (Friedman et al., 2010) was applied with its default values for the alpha and nfolds parameters (i.e., 1 and 10 respectively). For this function, the squared loss (i.e., mse in cv.glmnet) was used for cross-validation and its associated lambda.min parameter was used as the optimal lambda for prediction.

To evaluate the genomic predictive ability of the four methods, cross-validations were performed by sampling randomly a training and a target population 100 times for each case among the 60 situations. For each random sampling the sizes of the training and target sets were, respectively, two-thirds and one-third times the size of the total population. The Pearson correlation, between the predicted genetic values and the observed phenotypes for the target set, was taken as a measure of relative prediction accuracy (RPA). Indeed, true prediction accuracy (TPA) can be attained only if the true genetic values for the target set are available. The signal-to-noise ratio (SNR) (Czanner et al., 2015) for each method, with respect to each target set, was calculated as the sample variance of the predicted genetic values over the sample variance of the estimated residuals associated to the target phenotypes. Note that the SNR is related to genomic based heritabilities (De los Campos et al., 2013b; Janson et al., 2015). However, there are many different definitions of heritability (Janson et al., 2015) and these are different according to each studied method here. Hence we report only the estimated SNR for each method.

## 3. Results

**Figure 1:**
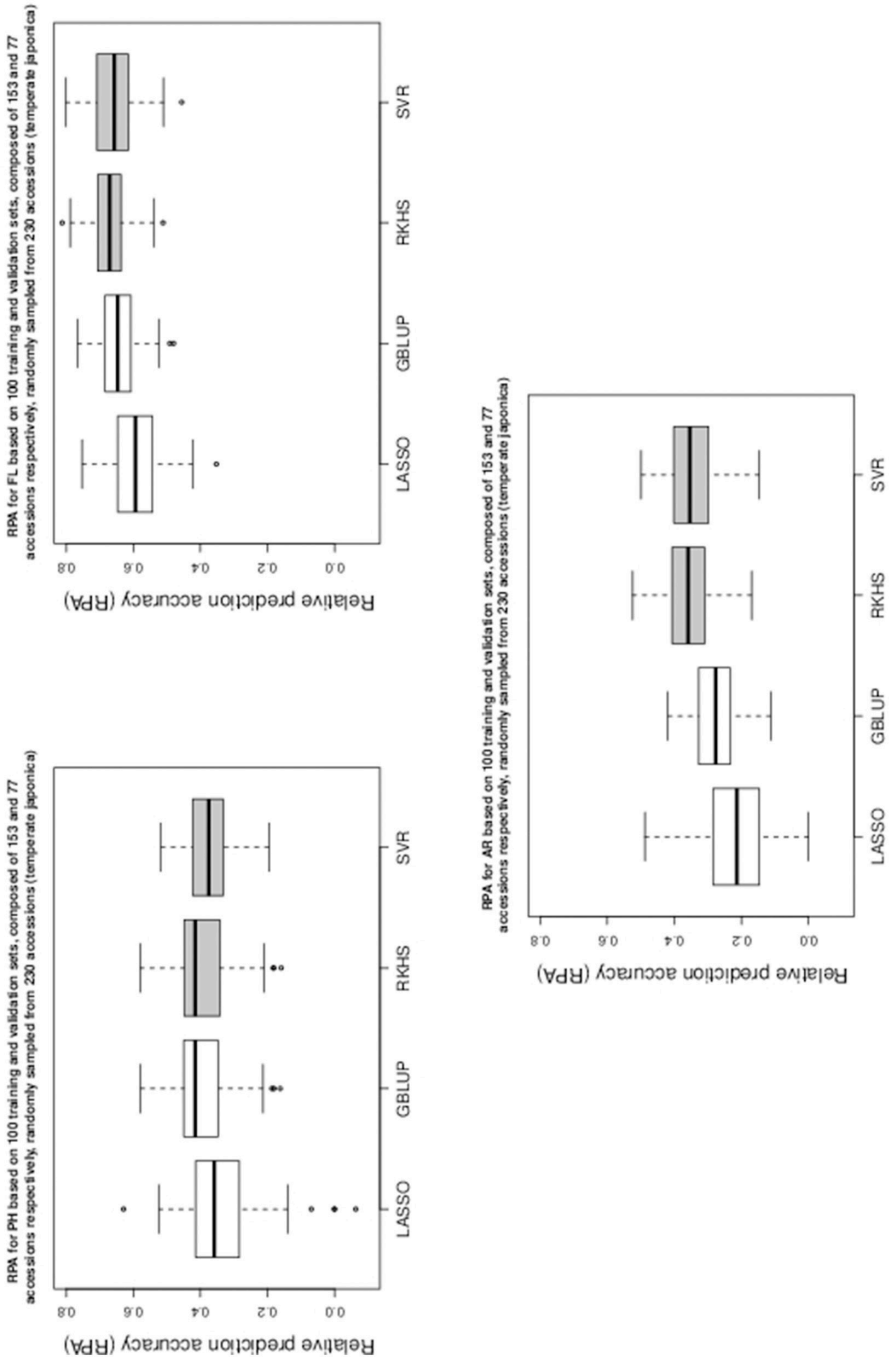
**Boxplots of RPA distributions associated to PH, FL, and AR for data set 1**.

**Figure 2:**
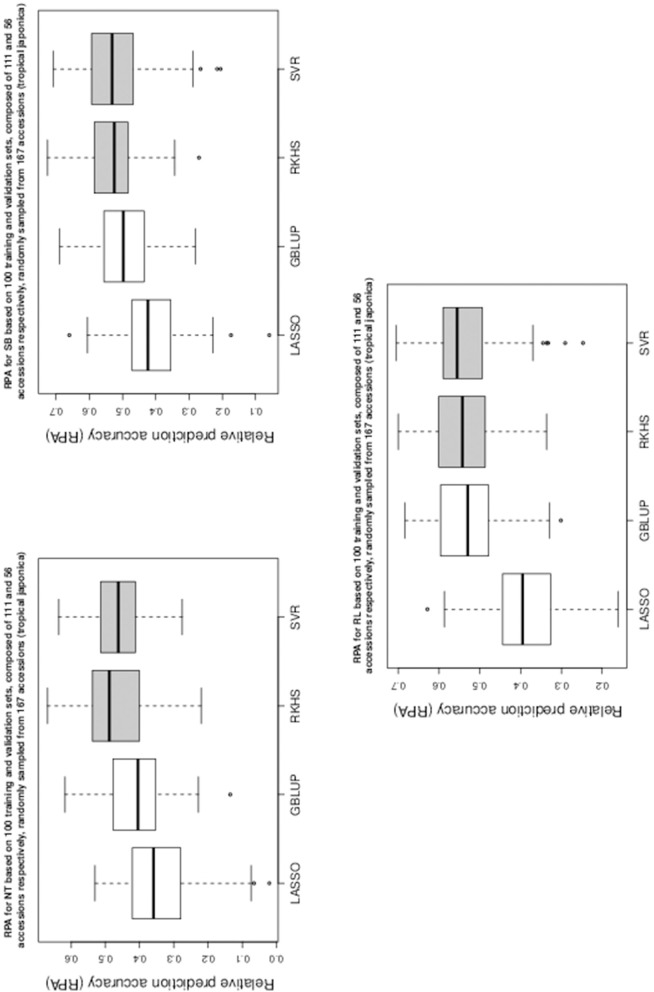
**Boxplots of RPA distributions associated to NT, SB, and RL for data set 2**.

**Figure 3:**
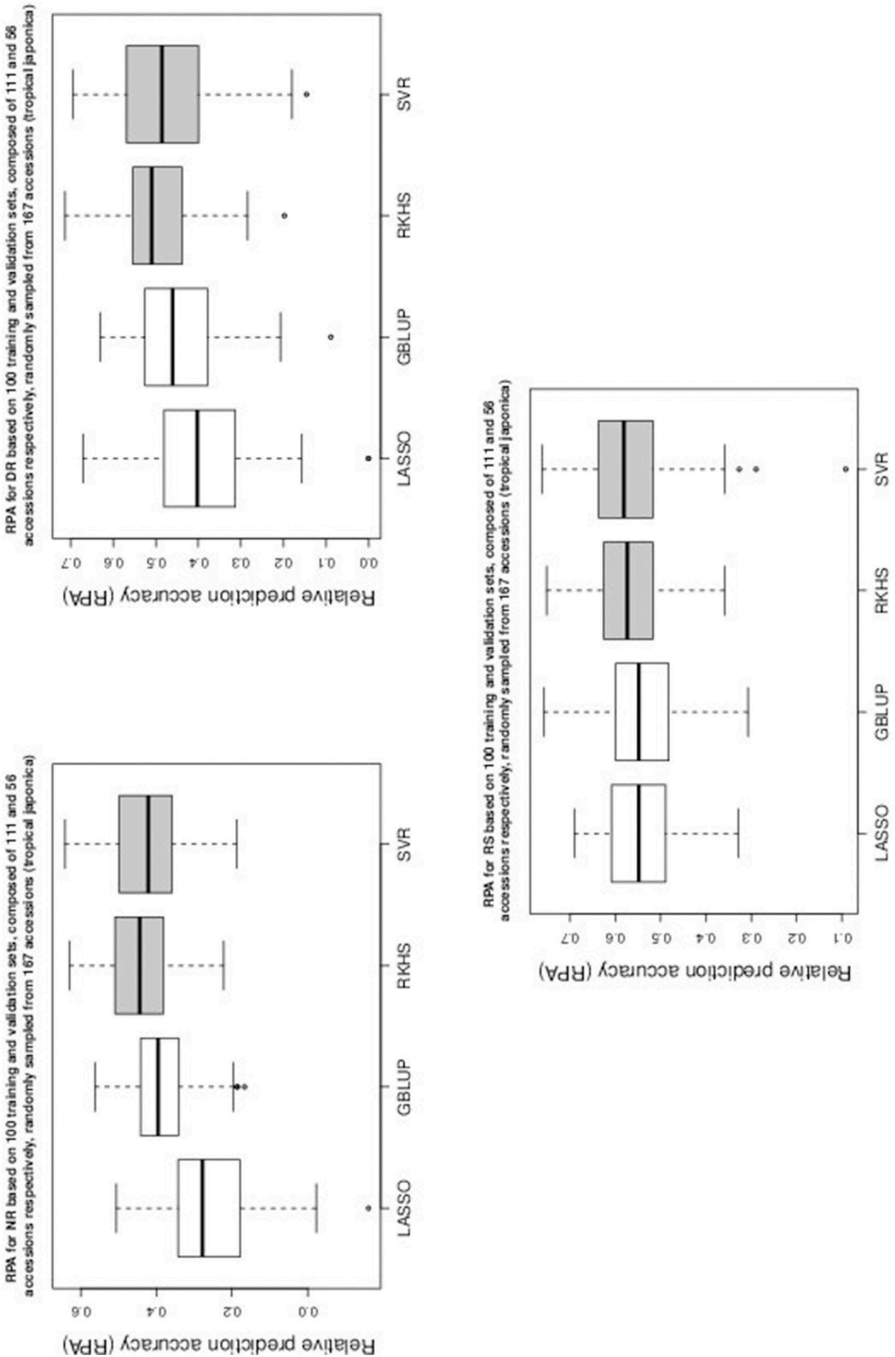
**Boxplots of RPA distributions associated to NR, DR, and RS for data set 2**.

**Figure 4:**
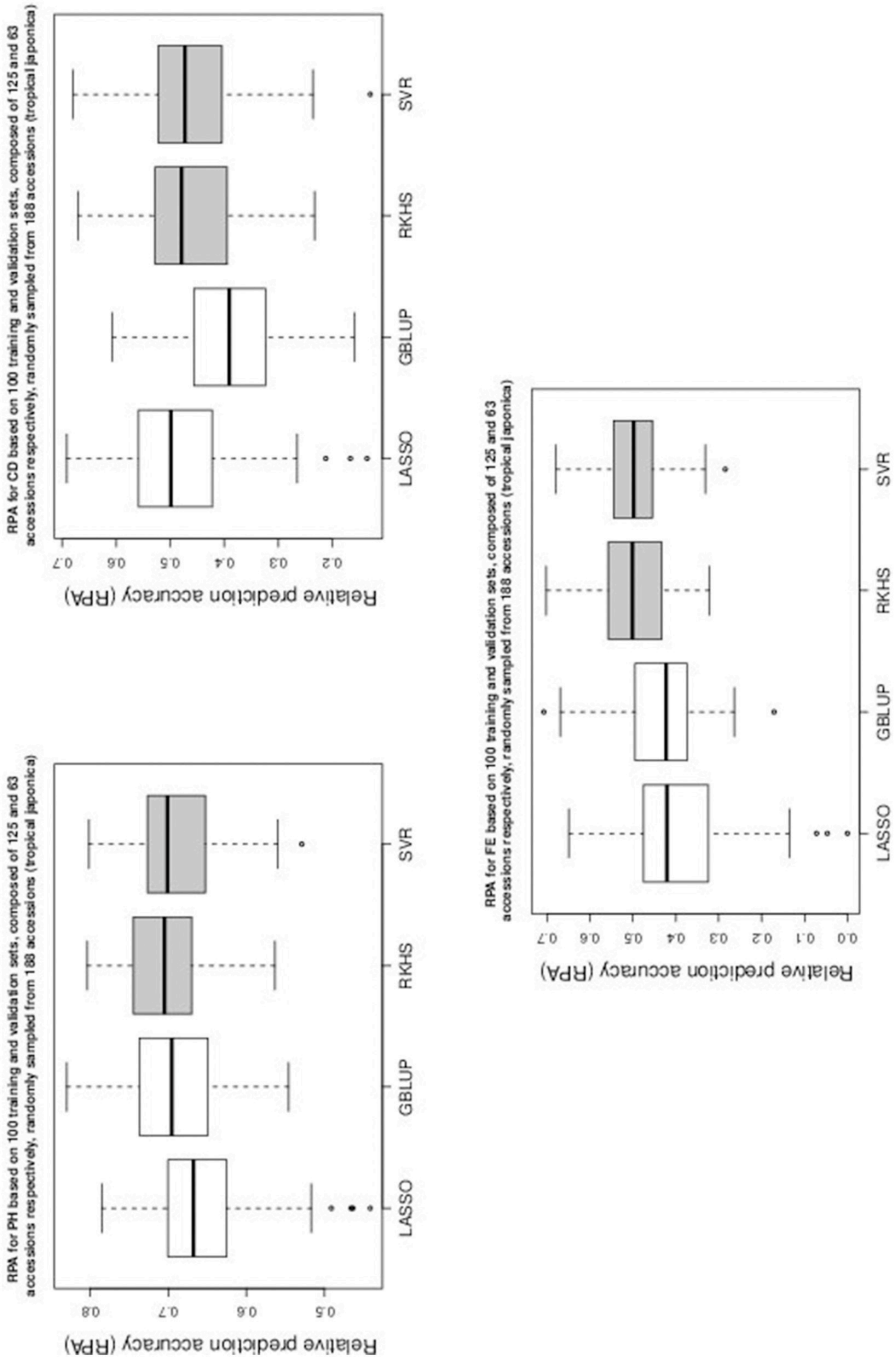
**Boxplots of RPA distributions associated to PH, CD, and FE for data set 3**.

**Figure 5:**
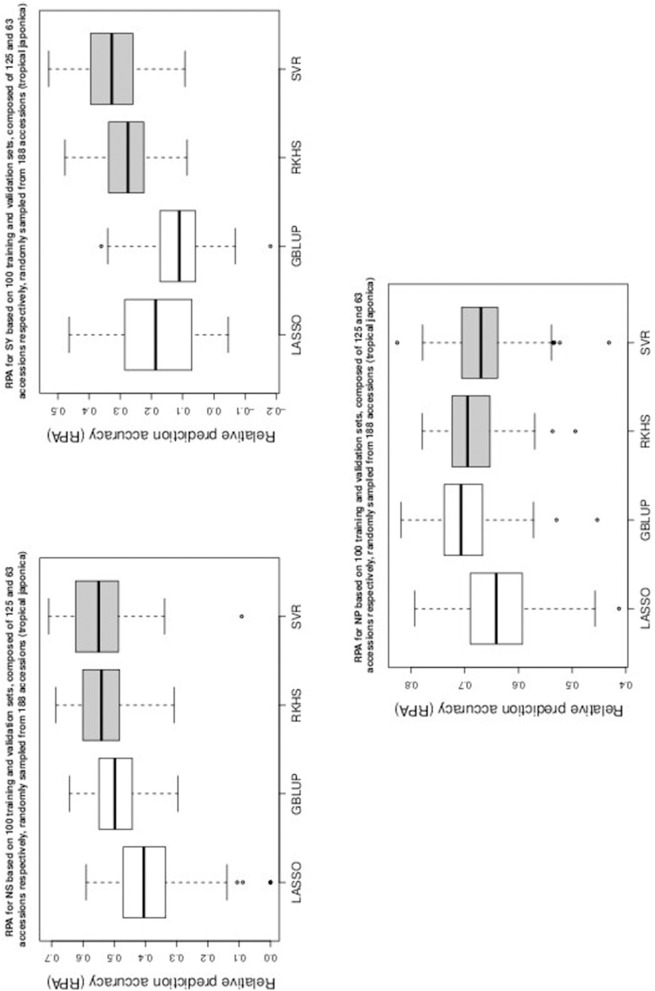
**Boxplots of RPA distributions associated to NS, SY, and NP for data set 3**.

Table 1 gives the RPA means with their associated standard errors and the SNR means for the 60 examined situations. The RPA standard errors and SNR means are given within parantheses and square brackets respectively. Figures 1–5 give the boxplots for the RPA distributions associated to the 60 studied cases.

**Table 1:** **RPA means with their associated standard errors within parantheses (.), and the SNR means within square brackets [.], for the 60 examined situations**.

**Data set**	**Trait**	**Method**			
		**LASSO**	**GBLUP**	**RKHS regression**	**SVR**
Data set 1	PH	0.34 (0.11) [0.11]	0.40 (0.08) [0.14]	0.40 (0.08) [0.16]	0.37 (0.07) [0.21]
230 accessions	FL	0.59 (0.07) [0.42]	0.65 (0.06) [0.93]	0.67 (0.06) [0.73]	0.66 (0.07) [0.75]
22691 SNP	AR	**0.21** (0.11) [0.10]	**0.27** (0.07) [0.35]	**0.35** (0.07) [0.12]	**0.35** (0.08) [0.20]
	NT	**0.34** (0.11) [0.25]	**0.41** (0.09) [0.59]	**0.47** (0.09) [0.24]	**0.46** (0.08) [0.32]
Data set 2	SB	**0.42** (0.09) [0.31]	**0.49** (0.09) [0.72]	**0.53** (0.09) [0.33]	**0.52** (0.10) [0.37]
167 accessions	RL	0.39 (0.09) [0.29]	0.53 (0.09) [0.39]	0.54 (0.08) [0.33]	0.54 (0.09) [0.40]
16444 SNP	NR	**0.25** (0.13) [0.16]	**0.39** (0.09) [0.31]	**0.44** (0.09) [0.17]	**0.42** (0.09) [0.31]
	DR	**0.39** (0.12) [0.29]	**0.45** (0.11) [0.67]	**0.49** (0.10) [0.40]	**0.48** (0.11) [0.21]
	RS	0.55 (0.08) [0.38]	0.54 (0.09) [0.70]	0.57 (0.07) [0.45]	0.57 (0.10) [0.30]
	PH	0.66 (0.07) [0.85]	0.69 (0.06) [1.15]	0.70 (0.05) [0.90]	0.69 (0.06) [0.81]
Data set 3	CD	0.48 (0.11) [0.29]	0.39 (0.09) [0.58]	0.47 (0.09) [0.26]	0.46 (0.09) [0.38]
188 accessions	FE	**0.39** (0.12) [0.28]	**0.43** (0.10) [0.58]	**0.50** (0.09) [0.46]	**0.50** (0.08) [0.47]
38390 SNP	NS	**0.38** (0.12) [0.33]	**0.50** (0.08) [0.44]	**0.54** (0.08) [0.38]	**0.55** (0.09) [0.45]
	SY	**0.18** (0.13) [0.14]	**0.12** (0.09) [0.03]	**0.28** (0.09) [0.10]	**0.33** (0.10) [0.28]
	NP	0.64 (0.08) [0.85]	0.70 (0.06) [0.80]	0.68 (0.06) [0.62]	0.67 (0.06)[0.65]

As can be seen in Table 1 and in Figures 1–5, RKHS regression and SVR performed as well or better than LASSO and GBLUP for most situations. Furthermore, in Figures 1–5, RKHS regression and SVR gave RPA values strictly greater than 0, for all data sets and traits, compared to LASSO and GBLUP. Indeed, LASSO gave negative RPA values for PH and NR as can be seen in Figures 1, 3, respectively. In Figure 5, both LASSO and GBLUP gave negative RPA values for SY.

In these figures and in Table 1, the largest RPA mean differences between parametric and kernel methods can be seen for AR, NT, SB, NR, DR, FE, NS, and SY (see bold values in Table 1). For these traits, the RPA mean differences between the parametric and kernel methods varied between 0.03 and 0.21. The highest observed RPA mean difference of 0.21 was between SVR and GBLUP for SY. For CD, one can see in Table 1 that the RPA and SNR means for GBLUP were simultaneously lower and higher than those of the other methods. This can be explained by a poor consistency of GBLUP, with respect to the DGP for this trait, which leads to an over-estimation of the true SNR. For example, it was shown in the second subsection that the poor consistency of a linear model, due to over-fitting, could minimize substantially the estimated residual variance, thus leading to an over-estimation of the true SNR while inducing a poor predictive ability.

Among the kernel methods, RKHS regression was often more accurate than SVR although only little RPA mean differences can be observed between these methods in Table 1. On the other hand, GBLUP was often more accurate than LASSO for the parametric methods. As can be seen in Table 1 and in Figures 1–5, LASSO had a much lower predictive performance than the other methods for most traits. The average of the RPA means for each method, across all traits for the three data sets, were 0.51, 0.50, 0.46, and 0.41 for RKHS regression, SVR, GBLUP and LASSO respectively.

For each analyzed trait, RKHS regression was performed in a reasonable computation time. For example, the computation time of one particular cross-validation for NT was 2.03 s on a personal computer with 8 GB RAM. However, depending on the trait considered, the computation time for RKHS regression was either lower or higher than that for SVR. For example, the computation times associated to one cross-validation for NT were 2.99 and 2.03 seconds for SVR and RKHS regression respectively. However, the computation times associated to one cross-validation for RL were 2.25 and 3.32 seconds for SVR and RKHS regression respectively. This can be explained by the, well known, slow convergence properties of the EM algorithm in some situations (Naim and Gildea, 2012).

## 4. Discussion

### 4.1. Comparison of the genomic predictive abilities of LASSO, GBLUP, SVR and RKHS regression

Among all the compared methods, RKHS regression and SVR were regularly the most accurate methods for prediction followed by GBLUP and LASSO. On the other hand, LASSO was often the least accurate method for prediction. This can be explained by the fact that, for situations where *p* > *n*, the predictive performance of LASSO is regularly dominated by Ridge regression (i.e., GBLUP) when covariates are highly correlated (Tibshirani, 1996; Zou and Hastie, 2005). Moreover, Dalalyan et al. (2014) recently showed that the predictive performance of LASSO can be mediocre, irrespective of the choice of the tuning parameter, when covariates are moderately correlated. For SNP marker data it is common to have high numbers of moderately and highly correlated markers due to linkage disequilibrium. Furthermore, there was a limited number of accessions (i.e., *n*) for the three studied data sets. This may also explain the less accurate performance of LASSO. Indeed, it is well known that the number of non null coefficients for an estimated LASSO model is bounded by *min*(*n, p*) (Tibshirani, 2013). Hence, when *p* > *n*, the number of markers (i.e., covariates) selected as relevant will be bounded by the number of accessions which may be inconsistent with the DGP. Alongside, Onogi et al. (2015) reported that the estimation of parameters via REML for GBLUP could be problematic for small sample size. This was observed for CD in our study where the RPA and SNR means for GBLUP were simultaneously lower and higher than those of the other methods.

Nevertheless, the observed RPA mean differences between the studied methods were somehow incremental for the three data sets. This is most probably due to the fact that our measure of RPA is based on the correlation between observed phenotypes, which are noisy measurements *per se*, and predicted genetic values. Moreover, Gianola et al. (2014) pointed out that differences among methods can be masked by cross-validation noise. Simulation studies conducted by our team (work to be published), based on real data for four traits, indicate that differences between methods based on TPA are often much higher than those based on RPA for the same simulated data set. In other words, small differences in RPA can be an indicator of higher differences in TPA among methods. Results for these simulation studies, with the corresponding simulated data sets, are available at http://tropgenedb.cirad.fr/tropgene/JSP/interface.jsp?module=RICE as the “GS-RUSE.zip” folder.

Still, our results show that kernel methods can be more appropriate than conventional parametric methods for many traits with different genetic architectures. These results are consistent with those of many previous studies (Konstantinov and Hayes, 2010; Pérez-Rodríguez et al., 2012; Sun et al., 2012; Howard et al., 2014). With respect to Morota and Gianola (2014), our results also indicate that kernel methods will have higher predictive performance, than conventional parametric methods, for traits potentially having moderate to complex epistatic genetic architectures. For example, the large RPA mean differences for SY, between the studied parametric and kernel methods, is probably due to an epistatic genetic architecture associated to this trait as pointed out by Liu et al. (2006). The same reasoning can be applied to AR for which epistatic mechanisms might potentially be involved (Norton et al., 2010). In this study, SVR and RKHS regression had similar predictive abilities. However, one advantage of RKHS regression over SVR lies in the fewer number of parameters to be estimated, which can be automated quite easily. Thus, RKHS regression can be performed more easily than SVR by low experienced users. Indeed, as pointed out by Cherkassky and Ma (2004), SVR application studies are usually performed by “practitioners,” who have a good understanding of the SVM methodology, since the main issue in having good SVM models lies in the proper setting of the meta-parameters.

### 4.2. Comparison and connections between kernel methods and other methods in frequentist and Bayesian frameworks

In comparison with Howard et al. (2014), we did not compare the studied kernel methods to neural networks (NN). Nevertheless, these authors showed that NN did not perform better than these methods in their simulation study. As pointed out by Howard et al. (2014), it is well-known that NN can be prone to over-fitting which reduces predictive performance. Moreover, NN are plagued with the problem of local minima in comparison to support vector machines which are not (Smola and Schlkopf, 1998). Yet, connections between NN with a single layer of hidden units (i.e., neurons) and kernel machines exist (Cho and Saul, 2009). In our study we reviewed the equivalence between well-known regularized, mixed and Bayesian linear models. As a matter of fact, for parametric models where one can specify likelihoods, inferences from frequentist (i.e., maximum likelihood based approaches) and Bayesian procedures will be practically the same if *n* (i.e., number of accessions) becomes sufficiently large for a fixed *p*. This is a consequence of the so-called Bernstein-von Mises theorem (Ghosal et al., 1995; Ghosal, 1997). Moreover, we showed in this study that many parametric methods can be framed as kernel methods, with simple kernels, due to their equivalent primal and dual formulations. For instance, this was shown for Ridge regression, Bayesian Ridge regression, RR-BLUP and GBLUP which are mathematically equivalent methods for prediction.

Framing parametric methods as kernel machines with simple kernels has important implications in the sense that many kernel methods can be specified, and solved conveniently, in existing classical frequentist (e.g., embedding kernels in mixed models) and Bayesian frameworks. This was first pointed out by Gianola et al. (2006) and several following works (De los Campos et al., 2010; Endelman, 2011; Morota et al., 2013; Pérez and de los Campos, 2014) developed kernel methods in these frameworks. We also developed a simple and user-friendly R function within the mixed model framework, named Kernel_Ridge_MM.R, which allows users to perform RR-BLUP of marker effects, GBLUP and RKHS regression, with a Gaussian, Laplacian, polynomial or ANOVA kernel, in a reasonable computation time. In our study we used only the Gaussian kernel which performed well for RKHS regression. However, other kernels such as the polynomial or ANOVA kernel can be used. For instance, the ANOVA kernel was found to perform well in multidimensional regression problems (Hofmann et al., 2008). A modified version of this function named Tune_kernel_Ridge_MM.R, which allows users to tune the rate of decay parameter for RKHS regression based on K-folds cross validation, has also been developed for Windows, Linux and parallelized for HPC Linux clusters. Finally, an R package named KRMM, associated to these functions, has also been developed. The KRMM package and all scripts are publicly available at https://sourceforge.net/u/ljacquin/profile/. As conclusion, we recommend the use of kernel methods for genomic prediction, and selection, since the genetic architectures associated to quantitative traits are rarely known and can be very complex and complicated to model. Therefore, it seems more advisable to use data-driven prediction models, which can account for multiple orders of interaction, to assess the genetic merits of individuals.

## Author contributions

LJ wrote the manuscript, developed all scripts and the KRMM package. LJ performed the analyses. T-VC and NA read and approved the manuscript.

## Funding

This work was funded by Agropolis Foundation Grant n^o^ 1201-006.

### Conflict of interest statement

The authors declare that the research was conducted in the absence of any commercial or financial relationships that could be construed as a potential conflict of interest.

## Supplementary material

This file contains the proofs of lemmas in the main text.
