# Supplementary material for: A Unified and Comprehensible View of Parametric and Kernel Methods for Genomic Prediction with Application to Rice
Source: Front Genet. 2016 Aug 9;7:145. doi: 10.3389/fgene.2016.00145 (PMC4977290; doi:10.3389/fgene.2016.00145)
Supplement: Supplementary file 1 [file DataSheet1.pdf]

# *Supplementary Material*: A unified and comprehensible view of parametric and kernel methods for genomic prediction with application to rice

**Laval Jacquin*, Tuong-Vi Cao and Nourollah Ahmadi**

*Correspondence:
Author Name: Laval Jacquin
laval.jacquin@cirad.fr

To prove equation 3 we need the following definitions and lemmas. In what follows we write $\hat{f}_p = \hat{f}_p(X)$, where $\hat{f}_p(X) = X\hat{\beta}_{OLS}$, and $f^* = f^*(X)$ for the sake of clarity. We recall that $X$ is a $n \times p$ matrix and that $\hat{\beta}_{OLS} = (X'X)^{-1}X'Y$.

DEFINITION 1. $\mathbb{B}ias^2(\hat{f}_p) = || \, \mathbb{E}[\hat{f}_p] - f^* \, ||_2^2 = \sum_{i=1}^{n}\Big(\mathbb{E}[\hat{f}_p(X_i)] - f^*(X_i)\Big)^2$

DEFINITION 2. $\mathbb{V}ar(\hat{f}_p) = \mathbb{E}\Big[ \, || \, \hat{f}_p - \mathbb{E}[\hat{f}_p] \, ||_2^2 \, \Big] = \mathbb{E}\Bigg[ \sum_{i=1}^{n}\Big(\hat{f}_p(X_i) - \mathbb{E}[\hat{f}_p(X_i)]\Big)^2 \Bigg]$

LEMMA 1.

$$\mathbb{E}\Big[ \, || \, \hat{f}_p - f^* \, ||_2^2 \, \Big] = \mathbb{E}\Big[ \, || \, \hat{f}_p - \mathbb{E}[\hat{f}_p] \, ||_2^2 \, \Big] + || \, \mathbb{E}[\hat{f}_p] - f^* \, ||_2^2 = \mathbb{V}ar(\hat{f}_p) + \mathbb{B}ias^2(\hat{f}_p)$$

PROOF OF LEMMA 1. By definition, we have:

$$\begin{aligned}
\mathbb{E}\Big[ \, || \, \hat{f}_p - f^* \, ||_2^2 \, \Big] &= \mathbb{E}\Bigg[ \sum_{i=1}^{n}\Big(\hat{f}_p(X_i) - \mathbb{E}[\hat{f}_p(X_i)] + \mathbb{E}[\hat{f}_p(X_i)] - f^*(X_i)\Big)^2 \Bigg] \\
&= \mathbb{E}\Bigg[ \sum_{i=1}^{n} \Big[ \Big(\hat{f}_p(X_i) - \mathbb{E}[\hat{f}_p(X_i)]\Big)^2 \\
&\quad + 2\Big(\hat{f}_p(X_i) - \mathbb{E}[\hat{f}_p(X_i)]\Big)\Big(\mathbb{E}[\hat{f}_p(X_i)] - f^*(X_i)\Big) + \Big(\mathbb{E}[\hat{f}_p(X_i)] - f^*(X_i)\Big)^2 \Big] \Bigg]
\end{aligned}$$

$$= \mathbb{E}\left[\sum_{i=1}^{n}\Big(\hat{f}_p(X_i) - \mathbb{E}[\hat{f}_p(X_i)]\Big)^2\right]$$
$$+ 2\sum_{i=1}^{n}\Big(\underbrace{\mathbb{E}[\hat{f}_p(X_i)] - \mathbb{E}[\hat{f}_p(X_i)]}_{=0}\Big)\Big(\mathbb{E}[\hat{f}_p(X_i)] - f^*(X_i)\Big)$$
$$+ \sum_{i=1}^{n}\Big(\mathbb{E}[\hat{f}_p(X_i)] - f^*(X_i)\Big)^2$$
$$= \mathbb{E}\left[\sum_{i=1}^{n}\Big(\hat{f}_p(X_i) - \mathbb{E}[\hat{f}_p(X_i)]\Big)^2\right] + \sum_{i=1}^{n}\Big(\mathbb{E}[\hat{f}_p(X_i)] - f^*(X_i)\Big)^2$$

Note that $f^*$ is assumed to be deterministic, hence $\mathbb{E}[f^*] = f^*$ (i.e. $\mathbb{E}[f^*(X_i)] = f^*(X_i)$).

■

LEMMA 2. *Let $E_p$ be the subspace of $\mathbb{R}^n$ generated by the columns of $X$, i.e. $E_p = \Big\{X\beta\ ;\ \beta \in \mathbb{R}^p\Big\} = \Big\{\sum_{j=1}^{p}\beta_j X^{(j)}\ ;\ \beta_j \in \mathbb{R},\ X^{(j)} \in \mathbb{R}^n\Big\}$, and let $P_{E_p} = X(X'X)^{-1}X'$ be the orthogonal projection matrix on the subspace $E_p$, we have:*

$$\mathbb{B}ias^2(\hat{f}_p) = ||\ P_{E_p}f^* - f^*\ ||_2^2 \tag{2.1}$$

$$\mathbb{V}ar(\hat{f}_p) = \sigma^2_{\varepsilon^*}p \tag{2.2}$$

PROOF THAT $P_{E_p}$ IS AN ORTHOGONAL PROJECTOR. $P_{E_p}$ is characterized by $i)$ $\forall z \in \mathbb{R}^n$, $P_{E_p}z \in E_p$ and $ii)$ $\forall v \in E_p,\ \forall z \in \mathbb{R}^n$, $v \perp (z - P_{E_p}z)$. For $i)$, let $z \in \mathbb{R}^n$ we have

$$P_{E_p}z = X[\underbrace{(X'X)^{-1}X'z}_{\in\mathbb{R}^p}]$$

, which belongs to $E_p$ by definition. For $ii)$, let $v \in E_p$ (i.e. $v = X\beta$) and $< .,. >_{\mathbb{R}^n}$ denotes the inner product between two vectors in $\mathbb{R}^n$, we have

$$< v, z - X(X'X)^{-1}X'z > = < X\beta, z - X(X'X)^{-1}X'z >$$
$$= < \beta, X'(z - X(X'X)^{-1}X'z) > \text{ (property of the inner product)}$$
$$= < \beta, X'z - \underbrace{X'X(X'X)^{-1}}_{=Id}X'z > = 0$$

■

PROOF OF 2.1 FOR LEMMA 2. By definition we have $\mathbb{B}ias^2(\hat{f}_p) = ||\ \mathbb{E}[\hat{f}_p] - f^*\ ||_2^2$ where

$$\mathbb{E}[\hat{f}_p] = \mathbb{E}[\hat{f}_p(X)] = \mathbb{E}[X\hat{\beta}_{OLS}] = \mathbb{E}[X(X'X)^{-1}X'Y] = \mathbb{E}[P_{E_p}Y]$$
$$= P_{E_p}\mathbb{E}[Y] = P_{E_p}\mathbb{E}[\ f^* + \varepsilon^*\ ] = P_{E_p}\big[\ f^* + \underbrace{\mathbb{E}[\varepsilon^*]}_{=0}\ \big] = P_{E_p}f^*$$

■

Note that if $f^* \in E_{p^*} \subseteq E_p$ (i.e. $f^* \in E_p$) then $P_{E_p}f^* = f^* \Rightarrow \mathbb{B}ias^2(\hat{f}_p) = 0$

PROOF OF 2.2 FOR LEMMA 2. We have

$$\mathbb{V}ar(\hat{f}_p) = \mathbb{E}\Big[\ ||\ \hat{f}_p - \mathbb{E}[\hat{f}_p]\ ||_2^2\ \Big] = \mathbb{E}\Big[\ ||\ \hat{f}_p - P_{E_p}f^*\ ||_2^2\ \Big] = \mathbb{E}\Big[\ ||\ P_{E_p}Y - P_{E_p}f^*\ ||_2^2\ \Big]$$
$$= \mathbb{E}\Big[\ ||\ P_{E_p}(Y - f^*)\ ||_2^2\ \Big] = \mathbb{E}\Big[\ ||\ P_{E_p}\varepsilon^*\ ||_2^2\ \Big]$$

Since $||\ P_{E_p}\varepsilon^*\ ||_2^2$ is a scalar, we have $\mathbb{E}\Big[\ ||\ P_{E_p}\varepsilon^*\ ||_2^2\ \Big] = \mathbb{E}\Big[\ Tr[||\ P_{E_p}\varepsilon^*\ ||_2^2]\ \Big]$ where $Tr[.]$ is the trace operator. Knowing that the projection matrix $P_{E_p}$ is idempotent (and symmetric), we also have $||\ P_{E_p}\varepsilon^*\ ||_2^2 = \varepsilon^{*'}P'_{E_p}P_{E_p}\varepsilon^* = \varepsilon^{*'}P_{E_p}P_{E_p}\varepsilon^* = \varepsilon^{*'}P_{E_p}\varepsilon^*$. Hence, using this last result and the cyclic property of the trace we have $\mathbb{E}\Big[\ Tr[||\ P_{E_p}\varepsilon^*\ ||_2^2]\ \Big] = \mathbb{E}\Big[\ Tr[\varepsilon^{*'}P_{E_p}\varepsilon^*]\Big] = \mathbb{E}\Big[\ Tr[P_{E_p}\varepsilon^*\varepsilon^{*'}]\Big]$. Finally, since $E[.]$ and $Tr[.]$ are linear operators, and hence commute, we have $\mathbb{E}\Big[\ Tr[P_{E_p}\varepsilon^*\varepsilon^{*'}]\Big]$ $= Tr\Big[\ \mathbb{E}[P_{E_p}\varepsilon^*\varepsilon^{*'}]\Big] = Tr\Big[\ P_{E_p}\ \underbrace{\mathbb{E}[\varepsilon^*\varepsilon^{*'}]}_{=\mathbb{V}ar(\varepsilon^*)=I_n\sigma^2_{\varepsilon^*}}\ \Big] = \sigma^2_{\varepsilon^*}Tr[\ P_{E_p}] = \sigma^2_{\varepsilon^*}p$. The last equality simply comes from the fact that $Tr[\ P_{E_p}] = Tr[\ X(X'X)^{-1}X'] = Tr[\ X'X(X'X)^{-1}] = Tr[\ I_p] = p$ since $X$ is full column rank $p$.

■

LEMMA 3.

$$||Y - P_{E_p}f^*||_2^2 = ||Y - \hat{f}_p||_2^2 + ||P_{E_p}f^* - \hat{f}_p||_2^2 \tag{3.1}$$

$$\mathbb{E}\Big[||Y - \hat{f}_p||_2^2\Big] = ||P_{E_p}f^* - f^*||_2^2 + \sigma^2_{\varepsilon^*}(n-p) \tag{3.2}$$

PROOF OF 3.1 FOR LEMMA 3. We saw in lemma 2 that $\forall v \in E_p,\ \forall z \in \mathbb{R}^n,\ v \perp (z - P_{E_p}z)$. In other words $(z - P_{E_p}z) = (I - P_{E_p})z = P_{E_p^\perp}z$ belongs to $E_p^\perp$ which is known as the orthogonal complement of $E_p$. Note that $P_{E_p^\perp} = (I - P_{E_p})$ is the orthogonal projection matrix of $z$ onto $E_p^\perp$. Thus, for $Y \in \mathbb{R}^n$ we

have $Y - \hat{f}_p = Y - P_{E_p}Y = (I - P_{E_p})Y = P_{E_p^\perp}Y \in E_p^\perp$ and $\hat{f}_p - P_{E_p}f^* = P_{E_p}(Y - f^*) \in E_p$. Hence, using Pythagoras's theorem we have

$$||Y - P_{E_p}f^*||_2^2 = ||\underbrace{Y - \hat{f}_p}_{\in E_p^\perp} + \underbrace{\hat{f}_p - P_{E_p}f^*}_{\in E_p}||_2^2$$
$$\underset{Pythogoras's\ theorem}{=} ||Y - \hat{f}_p||_2^2 + ||P_{E_p}f^* - \hat{f}_p||_2^2$$

■

PROOF OF 3.2 FOR LEMMA 3. From the proof of 3.1 for lemma 3, we know that

$$||Y - \hat{f}_p||_2^2 = ||Y - P_{E_p}f^*||_2^2 - ||P_{E_p}f^* - \hat{f}_p||_2^2$$
$$\Rightarrow \mathbb{E}\Big[\ ||Y - \hat{f}_p||_2^2\ \Big] = \mathbb{E}\Big[||Y - P_{E_p}f^*||_2^2\ \Big] - \mathbb{E}\Big[||P_{E_p}f^* - \hat{f}_p||_2^2\ \Big]$$
$$= \mathbb{E}\Big[||Y - P_{E_p}f^*||_2^2\ \Big] - \sigma_{\varepsilon^*}^2 p$$

The last equality is a consequence of the proof of 2.2 for lemma 2, where it is shown that $\mathbb{V}ar(\hat{f}_p) = \mathbb{E}\Big[\ ||\ \hat{f}_p - \mathbb{E}[\hat{f}_p]\ ||_2^2\ \Big] = \mathbb{E}\Big[\ ||\ \hat{f}_p - P_{E_p}f^*\ ||_2^2\ \Big] = \sigma_{\varepsilon^*}^2 p$. For the first term on the right hand side of the last equality we have

$$\mathbb{E}\Big[||Y - P_{E_p}f^*||_2^2\ \Big] = \mathbb{E}\Big[||Y - f^* + f^* - P_{E_p}f^*||_2^2\ \Big]$$
$$= \mathbb{E}\Big[\ ||Y - f^*||_2^2\ + 2 < Y - f^*, f^* - P_{E_p}f^* > + ||f^* - P_{E_p}f^*||_2^2\ \Big]$$
$$= \mathbb{E}\Big[\ ||\varepsilon^*||_2^2\ \Big]\ + \underbrace{2 < \underbrace{\mathbb{E}[Y]}_{=f^*} - f^*, f^* - P_{E_p}f^* >}_{=0} + ||f^* - P_{E_p}f^*||_2^2$$
$$= \underbrace{\mathbb{E}\Big[\ \sum_{i=1}^{n} \varepsilon_i^{*2}\ \Big]}_{=\sum_{i=1}^n \mathbb{E}\left[\ \varepsilon_i^{*2}\ \right] = \sum_{i=1}^n \mathbb{V}ar\left[\ \varepsilon_i^*\ \right] \overset{i.i.d}{=} n\sigma_{\varepsilon^*}^2} + ||f^* - P_{E_p}f^*||_2^2$$
$$= n\sigma_{\varepsilon^*}^2 + ||f^* - P_{E_p}f^*||_2^2$$

Finally, putting everything together we have

$$\mathbb{E}\Big[\ ||Y-\hat{f}_p||_2^2\ \Big] = n\sigma^2_{\varepsilon^*}\ +\ ||f^*-P_{E_p}f^*||_2^2 - \sigma^2_{\varepsilon^*}p\ =\ ||P_{E_p}f^*-f^*||_2^2\ +\ \sigma^2_{\varepsilon^*}(n-p)$$

■

Using lemma 1,2 and 3 we can prove lemma 4 which corresponds to equation 3.

LEMMA 4.
$$\mathbb{E}\Big[\ ||\hat{f}_p-f^*||_2^2\ \Big] = \mathbb{E}\Big[\ ||Y-\hat{f}_p||_2^2\ \Big] + 2\sigma^2_{\varepsilon^*}p - n\sigma^2_{\varepsilon^*}$$

PROOF OF LEMMA 4. Combining lemma 1 and 2 gives:

$$\mathbb{E}\Big[\ ||\ \hat{f}_p-f^*\ ||_2^2\ \Big]\ =\ \sigma^2_{\varepsilon^*}p + ||\ P_{E_p}f^*-f^*\ ||_2^2\ \Leftrightarrow\ ||\ P_{E_p}f^*-f^*\ ||_2^2\ =\ \mathbb{E}\Big[\ ||\ \hat{f}_p-f^*\ ||_2^2\ \Big] - \sigma^2_{\varepsilon^*}p$$

Hence, replacing $||\ P_{E_p}f^*-f^*\ ||_2^2$ with $\mathbb{E}\Big[\ ||\ \hat{f}_p-f^*\ ||_2^2\ \Big] - \sigma^2_{\varepsilon^*}p$ in lemma 3 gives

$$\begin{aligned} \mathbb{E}\Big[\ ||Y-\hat{f}_p||_2^2\ \Big]\ &= \mathbb{E}\Big[\ ||\ \hat{f}_p-f^*\ ||_2^2\ \Big] - \sigma^2_{\varepsilon^*}p\ +\ \sigma^2_{\varepsilon^*}(n-p) \\ \Leftrightarrow \mathbb{E}\Big[\ ||\ \hat{f}_p-f^*\ ||_2^2\ \Big]\ &= \mathbb{E}\Big[\ ||Y-\hat{f}_p||_2^2\ \Big]\ + 2\sigma^2_{\varepsilon^*}p\ -\ \sigma^2_{\varepsilon^*}n \end{aligned}$$

■

LEMMA 5. *We have* $\hat{f}_p(X) = X\hat{\beta}_{OLS} = Y$ *when* $p \geq n$.

PROOF OF LEMMA 5. We have $\hat{f}_p(X) = X\hat{\beta}_{OLS} = X(X'X)^{-1}X'Y = P_{E_p}Y$. Since $X$ is full column rank $p$ ($\Rightarrow p \leq n$), the column vectors of $X$ form a basis of $\mathbb{R}^n$ when $p = n$. More precisely, we have $E_p = \mathbb{R}^n$ when $p \geq n$. In other words, any element of $\mathbb{R}^n$ can be generated by a linear combination of the column vectors of $X$ when $p \geq n$. Since $Y \in \mathbb{R}^n$ we have $\hat{f}_p(X) = P_{\mathbb{R}^n}Y = Y$ when $p \geq n$.

■

LEMMA 6. *More than one feature map can be associated to a unique kernel* $k$.

PROOF OF LEMMA 6. Assume that every kernel has a unique associated feature map. Without loss of generality take $p = 2$, i.e. $X_i = [X_i^{(1)}, X_i^{(2)}] \in \mathbb{R}^2$ for $i = 1, .., n$. For the quadratic kernel $k(X_i, X_j) =$

$(< X_i, X_j >_{\mathbb{R}^2})^2$ we have

$$\begin{aligned} k(X_i, X_j) &= (< X_i, X_j >_{\mathbb{R}^2})^2 \\ &= \ < \phi(X_i), \phi(X_j) >_{\mathbb{R}^3} \\ &= \ < \tilde{\phi}(X_i), \tilde{\phi}(X_j) >_{\mathbb{R}^4} \end{aligned}$$

where $\phi$ and $\tilde{\phi}$ are the following feature maps:

$$\begin{aligned} \phi(X_i) &= [\ (X_i^{(1)})^2,\ \sqrt{2}X_i^{(1)}X_i^{(2)},\ (X_i^{(2)})^2\ ] \in \mathbb{R}^3 \\ \tilde{\phi}(X_i) &= [\ (X_i^{(1)})^2,\ X_i^{(1)}X_i^{(2)},\ (X_i^{(2)})^2,\ X_i^{(2)}X_i^{(1)}\ ] \in \mathbb{R}^4 \end{aligned}$$

Hence we have a contradiction with the assumption that every kernel has a unique associated feature map. In other word, a kernel $k$ can have one or more than one associated feature map(s).

■

LEMMA 7. *The Gaussian kernel* $k(X_i, X_j) = e^{-h||X_i - X_j||_2^2}$ *is associated to an infinite-dimensional feature map.*

PROOF OF LEMMA 7. Without loss of generality take $h = 1$, then we have

$$\begin{aligned} e^{-\frac{1}{2}||X_i - X_j||_2^2} &= e^{-\frac{1}{2}(||X_i||_2^2 - 2<X_i,X_j> + ||X_j||_2^2)} \\ &= e^{-\frac{1}{2}||X_i||_2^2}\, e^{-\frac{1}{2}||X_j||_2^2}\, e^{<X_i,X_j>} = e^{-\frac{1}{2}||X_i||_2^2}\, e^{-\frac{1}{2}||X_j||_2^2} \sum_{k=0}^{+\infty} \frac{(< X_i, X_j >)^k}{k!} \end{aligned}$$

where the last equality comes from Taylor's expansion for $e^x$.

If $p = 1$ then we have $< X_i, X_j >^k = X_i^k \times X_j^k$ where $X_i, X_j \in \mathbb{R}$ and $e^{-\frac{1}{2}||X_i - X_j||_2^2}$ can be written as

$$e^{-\frac{1}{2}||X_i - X_j||_2^2} = \sum_{k=0}^{+\infty} e^{-\frac{1}{2}||X_i||_2^2} \frac{X_i^k}{\sqrt{k!}} \times e^{-\frac{1}{2}||X_j||_2^2} \frac{X_j^k}{\sqrt{k!}} = \phi(X_i).\phi(X_j)$$

where $\phi(X_i) = \left(e^{-\frac{1}{2}||X_i||_2^2},\ e^{-\frac{1}{2}||X_i||_2^2} \frac{X_i^1}{\sqrt{1!}},\ e^{-\frac{1}{2}||X_i||_2^2} \frac{X_i^2}{\sqrt{2!}},\ ....\right) = \left(e^{-\frac{1}{2}||X_i||_2^2} \frac{X_i^k}{\sqrt{k!}}\right)_{k=0,\dots,+\infty}$

If $p > 1$ then we have $< X_i, X_j >^k = \left(X_i^{(1)}X_j^{(1)} + X_i^{(2)}X_j^{(2)} + .... + X_i^{(p)}X_j^{(p)}\right)^k = \left(\sum_{l=1}^{p} X_i^{(l)}X_j^{(l)}\right)^k$ where $X_i, X_j \in \mathbb{R}^p$. Moreover, from the multinomial theorem we have

$$\left(\sum_{l=1}^{p} X_i^{(l)} X_j^{(l)}\right)^k = \sum_{k_1+k_2+..+k_p=k} \frac{k!}{k_1!k_2!...k_p!} (X_i^{(1)} X_j^{(1)})^{k_1} (X_i^{(2)} X_j^{(2)})^{k_2} ... (X_i^{(p)} X_j^{(p)})^{k_p}$$
$$= \sum_{k_1+k_2+..+k_p=k} \Big(\frac{k!}{k_1!k_2!...k_p!}\Big)^{\frac{1}{2}} (X_i^{(1)})^{k_1} (X_i^{(2)})^{k_2} ... (X_i^{(p)})^{k_p}$$
$$\times \Big(\frac{k!}{k_1!k_2!...k_p!}\Big)^{\frac{1}{2}} (X_j^{(1)})^{k_1} (X_j^{(2)})^{k_2} ... (X_j^{(p)})^{k_p}$$

Hence, $e^{-\dfrac{1}{2}||X_i - X_j||_2^2}$ can be written as

$$e^{-\dfrac{1}{2}||X_i - X_j||_2^2} = e^{-\dfrac{1}{2}||X_i||_2^2} e^{-\dfrac{1}{2}||X_j||_2^2} \sum_{k=0}^{+\infty} \frac{1}{k!} \sum_{k_1+k_2+..+k_p=k} \Big(\frac{k!}{k_1!k_2!...k_p!}\Big)^{\frac{1}{2}} (X_i^{(1)})^{k_1} (X_i^{(2)})^{k_2} ... (X_i^{(p)})^{k_p}$$
$$\times \Big(\frac{k!}{k_1!k_2!...k_p!}\Big)^{\frac{1}{2}} (X_j^{(1)})^{k_1} (X_j^{(2)})^{k_2} ... (X_j^{(p)})^{k_p}$$
$$= \sum_{k=0}^{+\infty} \sum_{k_1+k_2+..+k_p=k} e^{-\dfrac{1}{2}||X_i||_2^2} \frac{1}{\sqrt{k!}} \Big(\frac{k!}{k_1!k_2!...k_p!}\Big)^{\frac{1}{2}} (X_i^{(1)})^{k_1} (X_i^{(2)})^{k_2} ... (X_i^{(p)})^{k_p}$$
$$\times\, e^{-\dfrac{1}{2}||X_j||_2^2} \frac{1}{\sqrt{k!}} \Big(\frac{k!}{k_1!k_2!...k_p!}\Big)^{\frac{1}{2}} (X_j^{(1)})^{k_1} (X_j^{(2)})^{k_2} ... (X_j^{(p)})^{k_p}$$
$$= \phi(X_i).\phi(X_j)$$

where $\phi(X_i) = \left( e^{-\dfrac{1}{2}||X_i||_2^2} \frac{1}{\sqrt{k!}} \Big(\frac{k!}{k_1!k_2!...k_p!}\Big)^{\frac{1}{2}} (X_i^{(1)})^{k_1} (X_i^{(2)})^{k_2} ... (X_i^{(p)})^{k_p} \right)_{k=0,...,+\infty,\ k_1+k_2+..+k_p=k}$ ■
